# Comment on “New Alternatives for Autoimmune Disease Treatments: Physicochemical and Clinical Comparability of Biosimilar Etanercept”

**DOI:** 10.1155/2018/5047067

**Published:** 2018-01-30

**Authors:** Brian Hassett, Steven Vicik, Brian Fitzpatrick

**Affiliations:** ^1^Pfizer Biotech, Dublin, Ireland; ^2^Pfizer Biotech, Andover, MA, USA

In their publication, Miranda-Hernandez et al. 2016 [1] present data on the physicochemical and biological comparability of Infinitam® with the reference etanercept innovator product, Enbrel®. As part of a physicochemical similarity exercise (not comparability), the authors present data on glycan microheterogeneity using hydrophilic interaction ultra-performance-liquid-chromatography (HILIC-UPLC) on three batches of Enbrel and one batch of Infinitam (Figure 3 panel b of Miranda-Hernandez et al.). We have presented a reproduction of Figure 3 panel b as Figure 1, and, for clarity, have labelled each chromatogram (a–d) (top to bottom). Miranda-Hernandez et al. present the top two and the bottom chromatograms as different batches of Enbrel (Figures 1(a), 1(b), and 1(d)). The third chromatogram (Figure 1(c)) is presented as one batch of Infinitam. We found the results in chromatogram a to be inconsistent with historical Enbrel manufacturing experience.

The glycan profile in chromatogram a is presented as being that of Enbrel; however, this profile is markedly different from the other batches of Enbrel presented and contains glycan species not resolved in the other batches. It is Pfizer's contention that chromatogram a is that of the single batch of Infinitam, and chromatogram c may be the third batch of Enbrel which Miranda-Hernandez et al. have analysed.

Pfizer and Amgen analyse the glycan profile of every batch of Enbrel manufactured using a high-performance liquid chromatography (HPLC) method similar to the UPLC method applied by Miranda-Hernandez et al. Pfizer and Amgen have release specifications for the glycan variants and the profile of every batch is compared to the reference standard. A batch with an atypical glycan profile like that of chromatogram a would not meet these specifications and thus would not be released to the market. Over 2000 batches have been analysed to date, and the HPLC peaks for the glycan profile have been consistent in every batch for nine characteristic peaks, with the relative abundance of each falling within tightly defined upper and lower limits. Therefore, the observations of Miranda-Hernandez et al. are considered extremely surprising. The level of heterogeneity shown between the batches presented by Miranda-Hernandez et al. does not reflect Pfizer's historical observations of the batch-to-batch variations in the glycan profile of Enbrel based on 18 years of commercial manufacture. Although the glycan profile of Enbrel is routinely tested using HPLC rather than HILIC-UPLC, this method has demonstrated a high degree of consistency across all batches tested, where profiles are aligned with a product-specific reference standard. We therefore find it puzzling that the authors found one batch of Enbrel to vary so substantially in comparison to the other two tested.

We have recently reported our findings on the analytical characterization for three batches of Infinitam, which included glycan analysis using HPLC [2]. Pfizer has also analysed these three batches of Infinitam by HILIC-UPLC, a method similar to the UPLC method used by Miranda-Hernandez et al. (Figure 2). The results of Pfizer's UPLC analyses were similar to HPLC analyses for the same samples. Compared with Enbrel, additional species were found in all three Infinitam batches, and of particular note, the chromatograms we obtained by UPLC were similar to the chromatogram in Figure 1(a), as presented by Figure 3(b) of Miranda-Hernandez et al.

Pfizer has characterised these additional species in Infinitam using mass spectrometry and identified them as A3G3F and A3G3FS1. These glycan species are not resolved in Enbrel samples in our routine HPLC method or the UPLC method.

It is acknowledged that the Enbrel batches tested by Miranda-Hernandez et al. were acquired in the United States (US) where Amgen is the market authorization holder. Amgen and Pfizer both manufacture Enbrel using the same cell clone and the same manufacturing process. Pfizer and Amgen also have established business processes to ensure the consistency of Enbrel across the globe. A third party assessment of Enbrel sourced from the US and European Union recently concluded that the product from the two regions was indistinguishable [3].

The glycan profile of Enbrel has been consistent for more than 2000 batches over a production span of 18 years [4]. We therefore question the accuracy of the glycan chromatograms of Enbrel presented by Miranda-Hernandez et al. and would encourage the authors to present data on further batches of Infinitam in order to substantiate these initial observations and also provide a more meaningful assessment of the structural comparability with Enbrel. Although Miranda-Hernandez et al. provided evidence of some structural similarity with Enbrel, we have highlighted significant structural differences between Enbrel and Infinitam [2]. For this reason, we contend that the authors have not provided sufficient evidence in this publication to conclude that Infinitam is highly similar to Enbrel.

## Figures and Tables

**Figure 1:**
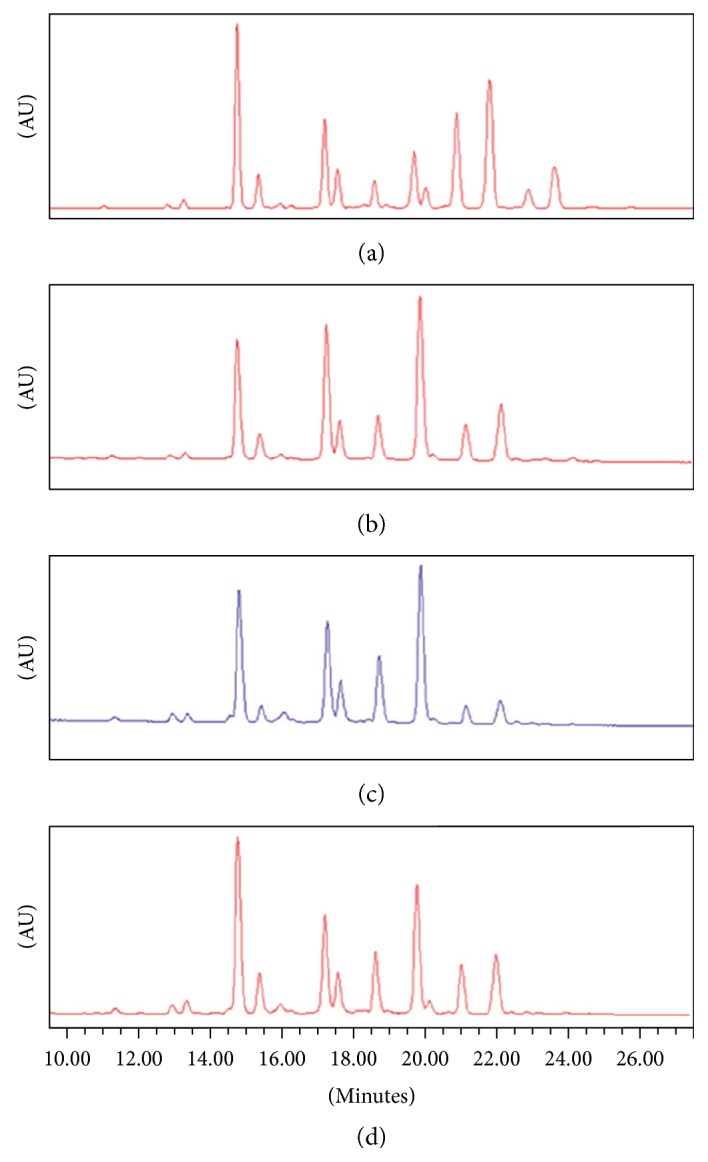
Figure 3(b) from Miranda-Hernandez et al. 2016 [1]. Glycan heterogeneity by HILI-UPLC of Infinitam (c) and the reference product (a, b, d). Chromatograms have been labelled (a–d) for this letter.

**Figure 2:**
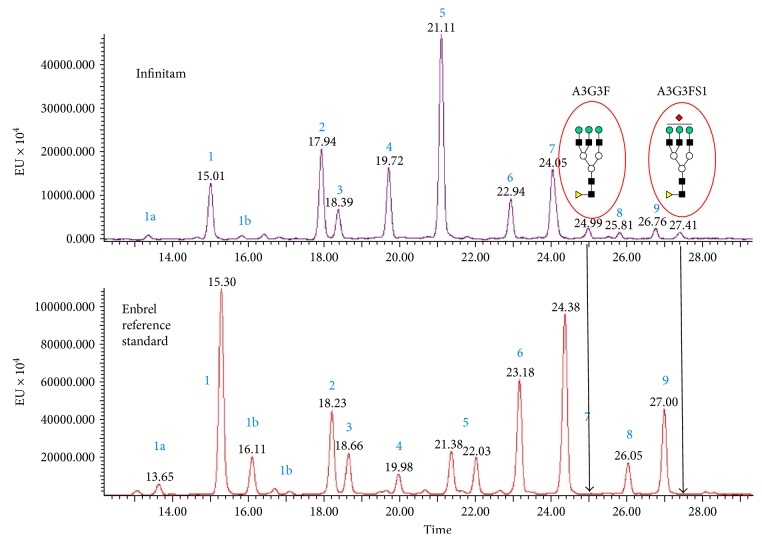
Pfizer's analysis of one representative batch of Infinitam by UPLC versus Enbrel reference standard. Note: All major glycans observed in Enbrel were identified in each of the three batches of Infinitam drug product. The overall abundance of grouped neutral (peaks 1–5) and sialylated (peaks 6–9) species was outside the commercial experience of Enbrel. Infinitam would not be regarded as comparable to Enbrel due to the presence of two glycan species in each batch of Infinitam which are not resolved in Enbrel (arrows show baseline for Enbrel).
